# Integrated Metabolomic and Transcriptomic Analyses Reveal the Differential Molecular Mechanisms Underlying Heat Stress Responses in Two *Pinellia ternata* Germplasms

**DOI:** 10.3390/genes17050512

**Published:** 2026-04-26

**Authors:** Guixia Shi, Zhen Yang, Guixiao La, Miao Huang, Yulong Zhao, Yaping Li, Tiegang Yang

**Affiliations:** 1 Institute of Chinese Herbal Medicines, Henan Academy of Agricultural Sciences, Zhengzhou 450002, China; sgx1668@hnagri.org.cn (G.S.); zju-l@163.com (G.L.); zhaoyulong2009@163.com (Y.Z.); liyaping0606@163.com (Y.L.); 2 Provincial Key Laboratory of Conservation and Utilization of Henan Traditional Chinese Medicine Resources, Zhengzhou 450002, China; 3 Xixian Agricultural Science Research Institute, Xinyang 464300, China; yangzhenkevin@163.com (Z.Y.); xxnksky@163.com (M.H.)

**Keywords:** *Pinellia ternata*, heat stress, basal thermotolerance, metabolome, transcriptome, molecular mechanisms

## Abstract

**Background**: *Pinellia ternata* is a major medicinal herb widely utilized in traditional medicine, but is sensitive to high temperature, which often triggers a severe “sprout tumble” phenomenon. **Methods**: To elucidate the molecular mechanisms of heat tolerance in *P. ternata*, we screened two contrasting germplasms: the heat-tolerant JBX1 and the heat-sensitive XBX4. In the present study, a combined analysis of physiology, transcriptome, and metabolome was performed on JBX1 and XBX4 under heat stress at 40 °C. **Results**: JBX1 exhibited significantly greater leaf thickness, higher basal chlorophyll content, more stable antioxidant enzyme activities, and lower oxidative damage than XBX4 under heat stress. Transcriptomically, JBX1 maintained elevated basal expression of genes encoding key enzymes in carbon fixation, amino acid metabolism, and phenylpropanoid biosynthesis, as well as those encoding heat shock transcription factors (HSFs), heat shock proteins (HSPs), and the thermosensor Thermo-With ABA-Response 1 (TWA1). Metabolomically, JBX1 accumulated higher levels of key primary metabolites, antioxidants, and protective phenylpropanoids under both control and heat conditions. Notably, a “polarity reversal” emerged in nitrogen metabolism, where core amino acids accumulated in JBX1 but were depleted in XBX4. Integrated analysis revealed a more coordinated gene–metabolite network in JBX1 involving the phenylpropanoid, ATP-binding cassette (ABC) transporter, and glutathione pathways. **Conclusions**: Our findings demonstrate that JBX1 possessed stronger basal thermotolerance, which is derived from coordinated establishment of higher constitutive metabolic reserves and efficient dynamic metabolic reprogramming. This study provides insights into the molecular mechanisms of heat stress in *P. ternata*.

## 1. Introduction

The 2023 IPCC Sixth Assessment Report pointed out that the land surface temperature was around 1.59 °C above 1850–1900 in 2011–2020, a warming primarily driven by human activities, and it is now highly probable that the global surface temperature will increase by at least 1.5 °C by 2050, a nearly unavoidable reality. Global warming will cause more extreme heat events, leading to a decline in staple crop yields (such as wheat and maize) by at least 5–10%, a trend that seriously conflicts with the doubling demand of global agricultural production by 2050 (https://www.ipcc.ch/, accessed on 13 September 2025). If a plant is exposed to environmental temperature that exceeds its optimum range by 5–10 °C, from a molecular perspective, heat stress can induce serious threats, including impaired photosynthetic efficiency, reduced respiratory enzyme activities, macromolecular damage, and a burst of reactive oxygen species (ROS) [1,2,3].

*Pinellia ternata* (Thunb.) Breit (known as Banxia in Chinese, Hange in Japanese, and Banha in Korean) is a perennial herbaceous plant belonging to the family Araceae and the genus *Pinellia*. As a traditional Chinese medicinal herb, *P. ternata* has a medical application history of over 2000 years in China [4]. Mature Pinelliae rhizome (PR) is produced from the underground tuber of *P. ternata*, serving as the medicinal portion in clinical applications and has been included in the national pharmacopeias of China, Japan, and Korea. Modern pharmacological studies indicate that *Pinellia*’s medicinal composition mainly consists of alkaloids, volatile oils, polysaccharides, organic acids, sterols, amino acids, and flavonoids, providing anti-emetic, anti-tussive, analgesic, anxiolytic, and anticancer effects [4,5,6]. According to data from China Customs, mainland China’s export trade volume of PR reached 15,552.63 tons, with a trade value of 227.57 million USD between 2011 and 2020 [6].

Under the combined effects of high light, high temperature, and drought stress, the aboveground parts of *P. ternata* frequently undergo lodging, wilting, and even death—a phenomenon commonly referred to as “sprout tumbling”. Therefore, high temperature and drought are the two major environmental factors causing sprout tumbling in production practice [7]. With continuous improvements in irrigation infrastructure, the impact of drought stress on *P. ternata* cultivation has been substantially mitigated. In contrast, heat stress has emerged as the predominant and intractable constraint on both tuber yield and medicinal quality [8,9]. *P. ternata* is mainly cultivated in the Chinese provinces of Gansu, Hubei, Hebei, and Sichuan [10]. In these regions, the summer daily maximum temperatures can occasionally reach 40 °C, with extreme heatwaves pushing soil surface temperatures above 42 °C. The summer months (July to August) represent a critical period for aboveground development; however, exposure to extreme heat during this window frequently triggers sprout tumbling, thereby shortening the vegetative phase and compromising both tuber yield and medicinal quality [8,9,10]. As a shade-loving sciophyte originating from understory habitats, *P. ternata* has an optimal growth temperature range of only 15–25 °C, rendering it exceptionally vulnerable to heat-induced disruption during this pivotal developmental stage. Exposure to extreme heat during this window frequently triggers sprout tumbling, abruptly terminating the development and severely compromising both yield and medicinal quality [8,9,10]. This heightened sensitivity to heat stress aligns with the findings of Fanourakis et al. [11], who reported that rising temperatures impose disproportionately severe constraints on horticultural crop production compared to other abiotic stressors. Notably, practical agronomic measures—such as shading, protected cultivation, or the strategic selection of higher-altitude planting sites—have been shown to effectively alleviate heat-induced sprout tumbling and improve both yield and quality [12,13]. Therefore, elucidating the molecular mechanisms underlying heat stress responses in *P. ternata* is essential not only for breeding thermotolerant varieties, but also for informing sustainable cultivation practices.

Previous findings have suggested that heat stress adversely damaged the structure of vascular tissues in the petioles and the homeostasis of protective enzymes, photosynthesis, and phytohormone systems in *P. ternata* [14,15,16,17,18]. In recent years, the process of identifying heat tolerance genes in *P. ternata* has been accelerated with omics technology. Comparative proteomic analysis was performed; underlying the response of *P. ternata* to heat stress, four primarily differentially expressed proteins were identified, including cytosolic class I small heat shock protein I/II, mitochondrial small heat shock protein, and glycine-rich RNA-binding protein [19]. Transcriptional responses to heat stress were conducted in *P. ternata*, indicating that many biological processes including photosynthesis, transmembrane transporter activity, plastid metabolism, plant hormone signal transduction, glutathione S-transferases synthesis, and heat shock proteins response were related to heat stress response [20,21]. Integrated transcriptome and miRNAome sequencing reported that MYB-like proteins and calcium-responsive transcription coactivators may play an integral role in heat stress resistance in *P. ternata* [22]. High temperature stress significantly induced the downregulation of DNA methylation level and upregulation of the full methylation rate in the *P. ternata* genome [23]. Meanwhile, it has been found that exogenous substances such as calcium ions, melatonin, and spermine can alleviate the damage of *P. ternata* under high temperatures [24,25]. Thus far, some resistance genes of *P. ternata* in response to high temperatures have been reported. *NAC66*, *PtsHSP17.2,* and *PtWRKY2* were positive transcriptional regulators in the heat tolerance of *P. ternata*, while *PtSAD* acted as a negative regulator [26,27,28,29]. Despite all this, the molecular mechanisms of *P. ternata* related to heat stress is still largely unknown.

To adapt to high-temperature stress, plants have evolved complex mechanisms, broadly categorized into basal thermotolerance and acquired thermotolerance. Acquired thermotolerance refers to the enhanced ability to withstand lethal temperatures following a non-lethal heat priming treatment, while basal thermotolerance is an inherent characteristic of plants that functions without the need for heat priming or heat acclimation processes [30]. As a temperature-sensitive plant, basal thermotolerance is particularly crucial for *P. ternata*, as it may help the plant survive brief episodes of extremely high temperatures. In this study, two genotypes of *P. ternata*—one heat-tolerant, JBX1, and one heat-sensitive, XBX4—were investigated using physiological assays, transcriptomics, and metabolomics. Results demonstrated that the stronger heat resistance of JBX1 is attributed to its high basal thermotolerance. Although related omics studies have been reported previously in *P. ternata*, this study is the first report to elucidate basal thermotolerance by integrating transcriptomic and metabolomic analyses using two contrasting germplasms with opposite heat responses.

## 2. Results

### 2.1. Phenotypic Analysis of JBX1 and XBX4 Under Heat Stress

**Figure 1:**
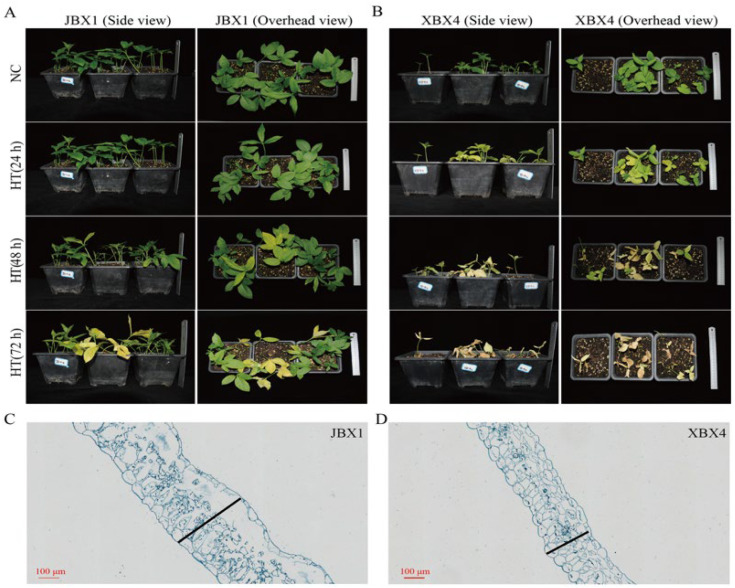
Effects of 72 h heat stress on the phenotypic characteristics of JBX1 and XBX4. (**A**) Phenotype of JBX1 under NC and HT conditions. (**B**) Phenotype of XBX4 under NC and HT conditions. (**C**,**D**) Cross sections of leaves from JBX1 and XBX4 under control conditions, visualized by paraffin sectioning.

**Figure 2:**
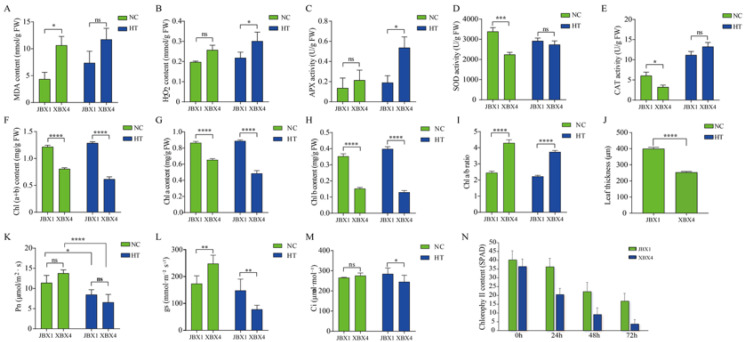
Physiological and biochemical responses of JBX1 and XBX4 under 8 h of heat stress. (**A**) MDA content; (**B**) H_2_O_2_ content; (**C**) APX activity; (**D**) SOD activity; (**E**) CAT activity; (**F**) Chl (a + b) content; (**G**) Chl a content; (**H**) Chl b content; (**I**) Chl a/b ratio; (**J**) leaf thickness (under control conditions); (**K**) *Pn*; (**L**) gs; (**M**) Ci; (**N**) chlorophyll content (SPAD). (**A**–**M**): Data are mean ± SD (*n* = 3); statistical significance was analyzed using two-way ANOVA. (**N**): Data are mean ± SD (n = 10). (* *p* < 0.05, ** *p* < 0.01, *** *p* < 0.001, **** *p* < 0.0001, ns: not significant).

To investigate the heat tolerance of *Pinellia ternata*, JBX1 and XBX4 were subjected to control (NC, 25 °C) and heat stress (HT, 40 °C for 72 h) conditions. The samples were designated as JBX1-NC, JBX1-HT, XBX4-NC, and XBX4-HT, respectively. We found that XBX4 showed yellowing leaves after 24 h of heat treatment, and the leaves were completely withered by 72 h. In contrast, JBX1 exhibited no significant change in leaf color at 24 h, slight yellowing at 48 h, and noticeable yellowing at 72 h (Figure 1A,B). Chlorophyll relative content was further assessed using a handheld SPAD meter. The results showed that the chlorophyll content of XBX4 declined more rapidly under heat stress, which was consistent with the visible leaf yellowing phenotype (Figure 2N). JBX1 demonstrated stronger heat resistance than XBX4.

### 2.2. Effects on Physiological and Biochemical Indicators of P. ternata Under Heat Stress

To assess the capacity for reactive oxygen species (ROS) scavenging in JBX1 and XBX4 under heat stress (40 °C, 8 h), we measured the levels of H_2_O_2_ and MDA as well as the activities of the major antioxidant enzymes, including ascorbate peroxidase (APX), superoxide dismutase (SOD), and catalase (CAT). The levels of malondialdehyde (MDA) and hydrogen peroxide (H_2_O_2_) serve as key indicators of oxidative damage in *P. ternata* under heat stress. Although basal H_2_O_2_ levels of JBX1 and XBX4 were similar under NC, JBX1 possessed significantly lower initial MDA levels than XBX4. Following HT, the two parameters increased in both JBX1 and XBX4. Notably, XBX4 displayed a significantly sharper rise in H_2_O_2_ and higher MDA levels compared to JBX1 (Figure 2A,B). The results of nitroblue tetrazolium (NBT) staining showed markedly darker and more extensive blue precipitates in the XBX4 leaves than JBX1, confirming a higher oxidative burden (Supplementary Figure S1). These data indicate that JBX1 maintains superior redox homeostasis and membrane integrity, likely due to its robust antioxidant capacity, which effectively mitigates heat-triggered ROS bursts.

Compared to XBX4, JBX1 exhibited higher basal SOD (*p* < 0.001) and CAT (*p* < 0.05) activities under NC, but lower APX activity. After heat stress, SOD activity in XBX4 showed an upward trend. Conversely, JBX1 maintained a relatively stable profile with only a slight reduction in SOD activity. CAT and APX activities were upregulated in response to heat stress in both genotypes (Figure 2C–E). However, the magnitude of induction was markedly more pronounced in XBX4 than in JBX1, indicating that XBX4 experienced more severe physiological fluctuations and was forced to mobilize its antioxidant pool more aggressively to counteract the heat-induced ROS burst.

In terms of photosynthetic capacity, JBX1 exhibited significantly higher basal chlorophyll content (chlorophyll a + b), chlorophyll a (Chl a), and chlorophyll b (Chl b) than XBX4 under NC (*p* < 0.0001). Following HT, while both materials exhibited a decline in chlorophyll levels, JBX1 consistently maintained significantly higher levels compared to XBX4 (Figure 2F–H). Notably, the Chl a/b ratio in XBX4 remained significantly higher than that in JBX1 under both NC and HT (Figure 2I). Interestingly, the net photosynthetic rate (*Pn*) of JBX1 was lower than that of XBX4 under NC, while JBX1 demonstrated superior photosynthetic resilience, characterized by a significantly slower reduction in *Pn* following heat exposure (Figure 2K). For stomatal conductance (gs): while XBX4 exhibited slightly higher gs under normal conditions, it underwent a drastic reduction under HT, falling significantly below the levels of JBX1 (Figure 2L). Under HT, the intercellular CO_2_ concentration (Ci) of XBX4 decreased significantly (Figure 2M). Notably, JBX1 leaf thickness significantly exceeded that of XBX4 (399 µm vs. 253 µm; Figure 1C,D and Figure 2J).

### 2.3. Post-Processing Quality Control and Data Reliability Assessment in Transcriptome of P. ternata Under Heat Stress

To explore the molecular mechanisms underlying the physiological differences between JBX1 and XBX4, RNA sequencing (RNA-Seq) was performed on leaves from both germplasms under NC and HT conditions (40 °C for 8 h). An average of 6.35 Gb of clean data was obtained per sample (Supplementary Table S1). The average percentage of Q30 bases for all samples was >95.90%, and the GC content averaged 52.45%, indicating high sequencing reliability. Approximately 79.79% of clean reads were successfully mapped to the *P. ternata* reference genome (Supplementary Table S1).

The Pearson correlation coefficient (PCC) and principal component analysis (PCA) were employed to evaluate the consistency of biological replicates and the relationships between treatment groups. The PCA results showed that the four groups (JBX1-NC, JBX1-HT, XBX4-NC, and XBX4-HT) were clearly separated in the PC1 × PC2 score plot, which accounted for 32.45% and 26.14% of the total variance, respectively. Notably, a distinct grouping trend was observed between JBX1 and XBX4, suggesting significant inherent and stress-induced differences in their transcriptomic landscapes (Supplementary Figure S2A). Pearson correlation analysis (Supplementary Figure S2B) showed that intra-group biological replicates exhibited high correlation (r > 0.94; e.g., JBX1-NC: 0.941~0.998; XBX4-HT: 0.998~0.999), however, the inter-group correlation was considerably lower (r = 0.707~0.953), demonstrating high technical reproducibility. Consistently, samples with identical treatments clustered together by hierarchical clustering analysis (Supplementary Figure S2C). Uniformity in sequencing depth and expression profiles among groups further validated the data for subsequent differential expression analysis (Supplementary Figure S2D).

### 2.4. Analysis of Relevant Gene Differences in P. ternata Under Heat Stress

**Figure 3:**
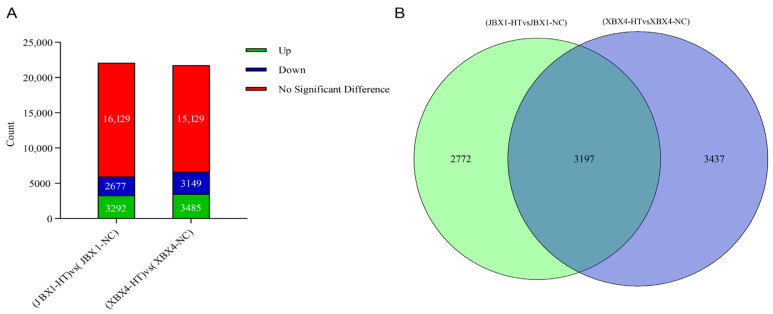
Characterization of DEGs in JBX1 and XBX4 under heat stress (40 °C for 8 h). (**A**) Numbers of upregulated (green) and downregulated (blue) DEGs in the JBX1-HT vs. JBX1-NC and XBX4-HT vs. XBX4-NC comparisons. (**B**) Venn diagram analysis of the DEGs of JBX1 and XBX4.

Differentially expressed genes (DEGs) (JBX1-HT vs. JBX1-NC, XBX4-HT vs. XBX4-NC) were identified using a threshold of |log_2_ (fold change)| > 1 and FDR < 0.05. The results showed that XBX4 recruited more DEGs (6634) compared to JBX1 (5969), suggesting that it underwent more extensive transcriptomic reprogramming. A total of 3197 DEGs were commonly expressed in both germplasms; 2772 DEGs were specifically regulated in JBX1, whereas 3437 DEGs were unique to XBX4 (Figure 3). The reliability and accuracy of RNA-Seq were confirmed by the real-time quantitative PCR (qRT-PCR) analysis of nine genes, including *PtGene26952*, *PtGene19732*, *PtGene40223*, *PtGene00331*, *PtGene00927*, *PtGene19099*, *PtGene39465*, *PtGene70858*, and *PtGene26973* (Supplementary Figure S3). Expression patterns of the nine genes by qRT-PCR were highly consistent with the relative transcript levels obtained from RNA-Seq. Thus, the RNA-Seq data generated in this study are reliable.

**Figure 4:**
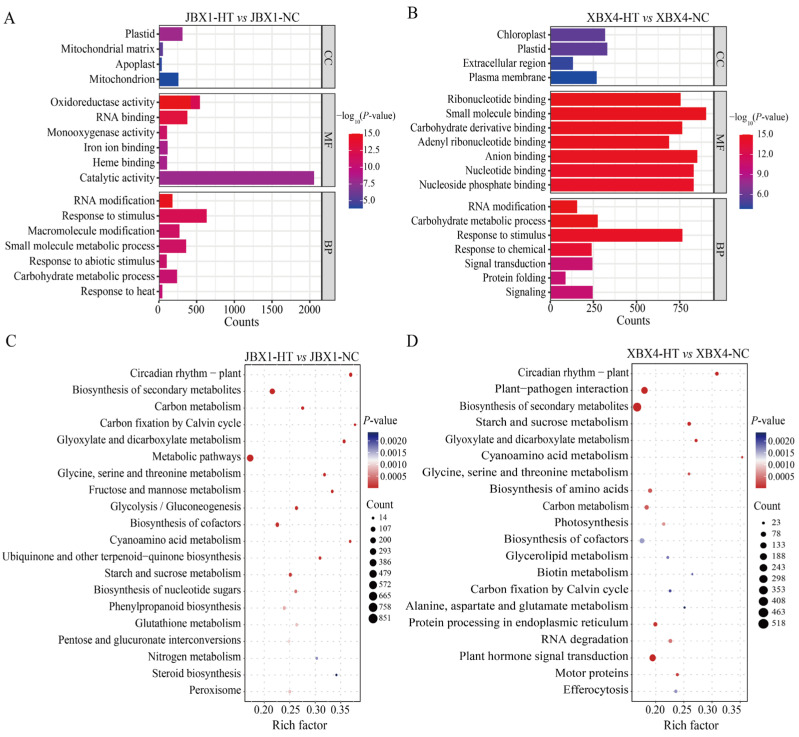
GO and KEGG enrichment analysis of DEGs under heat stress. (**A**,**C**) GO enrichment (**A**) and KEGG pathway enrichment (**C**) of DEGs in JBX1. (**B**,**D**) GO enrichment (**B**) and KEGG pathway enrichment (**D**) of DEGs in XBX4.

To explore the biological functions and metabolic pathways associated with the heat stress response in *P. ternata*, Gene Ontology (GO) and Kyoto Encyclopedia of Genes and Genomes (KEGG) enrichment analyses of the DEGs were performed. In both JBX1 and XBX4, the DEGs were significantly enriched in biological processes such as “response to stimulus”, “RNA modification”, and “carbohydrate metabolic process”. In the cellular component category, “plastid” was the dominant CC term. In the molecular function category, “catalytic activity” in JBX1, along with “small molecule binding” and “anion binding” in XBX4, were significantly enriched (Figure 4A,B).

KEGG pathway enrichment revealed that the DEGs in JBX1 were predominantly mapped to pathways including “carbon fixation by Calvin cycle”, “phenylpropanoid biosynthesis,” and “nitrogen metabolism”, indicating maintained energy homeostasis and structural integrity (Figure 4C). Unlike JBX1, XBX4 showed significant enrichment in “plant hormone signal transduction” and “protein processing in endoplasmic reticulum”, reflecting intensive stress signaling and cellular damage repair mechanisms (Figure 4D).

### 2.5. Comparative Analysis of Differentially Expressed Transcription Factors (DETFs)

**Figure 5:**
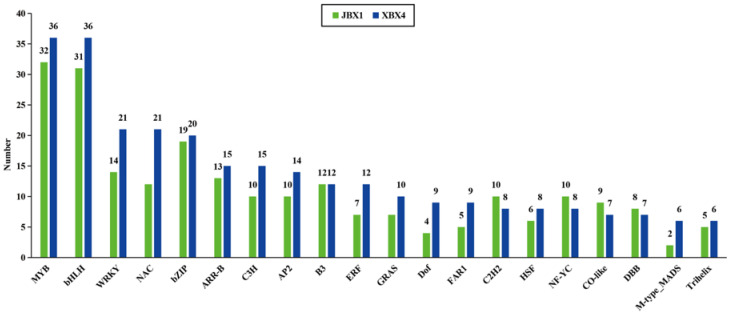
Differentially expressed transcription factors (TFs) in JBX1 and XBX4 under heat stress.

Transcription factors (TFs) play a pivotal role in plant defense against abiotic stress. We analyzed the distribution of DETFs in JBX1 and XBX4. A total of 20 TF families, including MYB, bHLH, WRKY, and NAC, were identified. Consistent with the global DEGs trend, XBX4 recruited a higher number of DETFs compared to JBX1 (e.g., 36 MYBs and 36 bHLHs in XBX4 vs. 32 MYBs and 31 bHLHs in JBX1) (Figure 5). This suggests that XBX4 undergoes a more drastic and potentially disordered transcriptional reprogramming by 40 °C heat stress.

**Figure 6:**
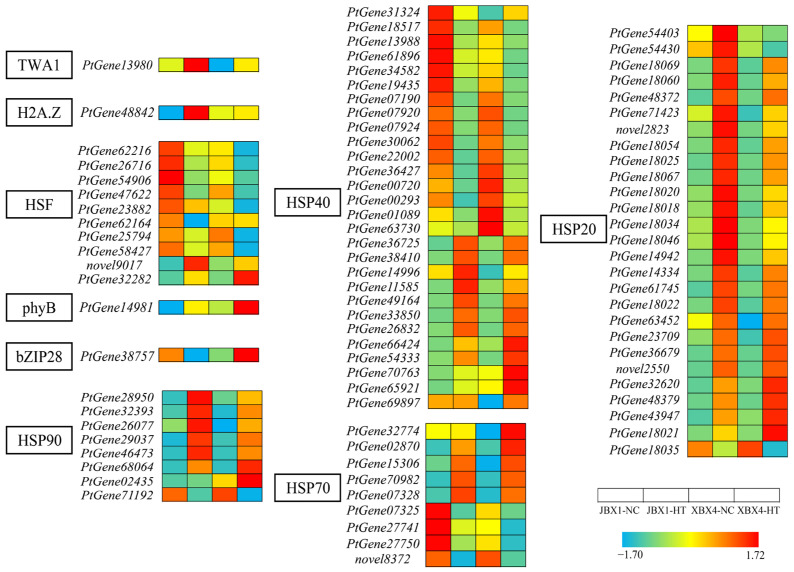
Heatmap of selected heat stress-related DEGs in JBX1 and XBX4. Genes shown include *HSF*, *HSP*, *TWA1*, *PHYB*, *H2A.Z*, and *bZIP28* family members. Color scale represents Z-score normalized expression (red: high; blue: low).

Heat shock transcription factor (HSF) is a core transcription factor in the regulatory network of heat stress responses. From the DEGs of JBX1 and XBX4, we screened a total of 10 *HSF* genes after heat stress, among them, the expression of seven *HSF* genes was suppressed in both materials. As shown in Figure 6, under NC, the expression levels of the majority of *PtHSF* genes (e.g., *PtGene62216*, *PtGene26716*, and *PtGene54906*) in JBX1 were higher than those in XBX4. We also identified many *Heat Shock Protein* (*HSP*) genes among the DEGs. Notably, nearly all members of the *HSP90* and *HSP20* subfamily genes were strongly induced by heat stress. Similar to the patterns observed for *HSF* genes, these *HSP* genes exhibited significantly higher expression levels in JBX1-NC than in XBX4-NC. In contrast, genes belonging to the HSP70 and HSP40 families exhibited divergent expression patterns, individual genes being either up- or downregulated. Furthermore, several key thermosensing and signaling components were also differentially regulated. The genes of the thermosensor *TWA1*, the histone variant *H2A.Z*, and the molecular thermometer *phytochrome B* (*phyB*) were all upregulated upon heat exposure, with JBX1 showing a stronger response (Figure 6). The gene encoding bZIP28 (a basic leucine zipper transcription factor), a hallmark sensor of endoplasmic reticulum (ER) stress, was downregulated in JBX1 but significantly upregulated in XBX4, implying that XBX4 suffered severe proteotoxic stress and protein misfolding.

### 2.6. Metabolomic Responses of P. ternata Under Heat Stress

Following the same protocol as for transcriptome analysis, both JBX1 and XBX4 were subjected to heat stress at 40 °C for 8 h and subsequently analyzed with non-targeted metabolomics. Principal component analysis (PCA) was initially performed to evaluate the overall variance among the four treatment groups. The PCA score plot revealed a clear separation among the four groups along the coordinates defined by PC1 (contributing 37.4%) and PC2 (contributing 13.7%), indicating significant differences in metabolite profiles between the treatment groups (Supplementary Figure S4A). Meanwhile, the spatial distribution of the two control groups was distinct, suggesting inherent differences in their basal metabolism. The clustering heatmap results were highly consistent with the PCA analysis, clearly segregating the samples into four distinct clusters (Supplementary Figure S4B).

**Figure 7:**
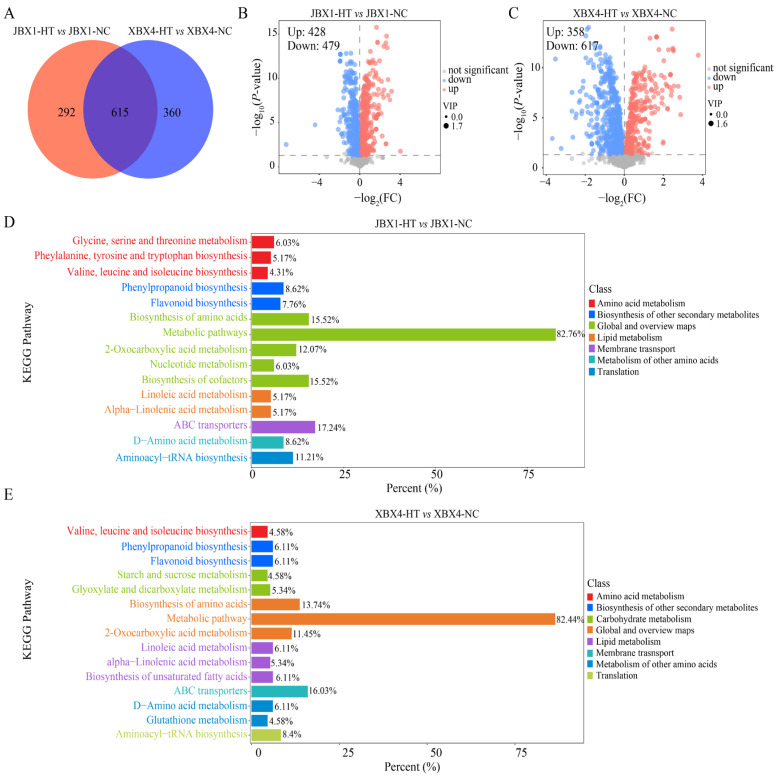
Metabolomic analysis of DAMs in JBX1 and XBX4 under heat stress (40 °C for 8 h). (**A**) Venn diagram analysis of DAMs in JBX1 and XBX4. (**B**,**C**) Volcano plots of DAMs in JBX1 (**B**) and XBX4 (**C**). (**D**,**E**) KEGG pathway enrichment analysis of DAMs in JBX1 (**D**) and XBX4 (**E**).

Differentially accumulated metabolites (DAMs) were identified using the thresholds of variable importance in projection (VIP) ≥ 1 and *p* < 0.05. In XBX4, a total of 975 DAMs were detected (358 upregulated and 617 downregulated) and 907 DAMs were identified (428 upregulated and 479 downregulated) in JBX1 (Figure 7B,C). Venn diagram analysis showed that 615 DAMs were commonly regulated in both materials, representing the core metabolic response to heat stress in *P. ternata*. A total of 292 metabolites were uniquely altered in JBX1, while 360 were specific to XBX4 (Figure 7A).

The DAMs were analyzed for KEGG enrichment (Figure 7D,E). Similar functional signatures were observed between the two materials. The DAMs identified in both JBX1 and XBX4 exhibited a marked enrichment in pathways such as ATP-binding cassette (ABC) transporter, biosynthesis of amino acids, phenylpropanoid biosynthesis, and flavonoid biosynthesis.

### 2.7. Combined Analyses of DEGs and DAMs

**Figure 8:**
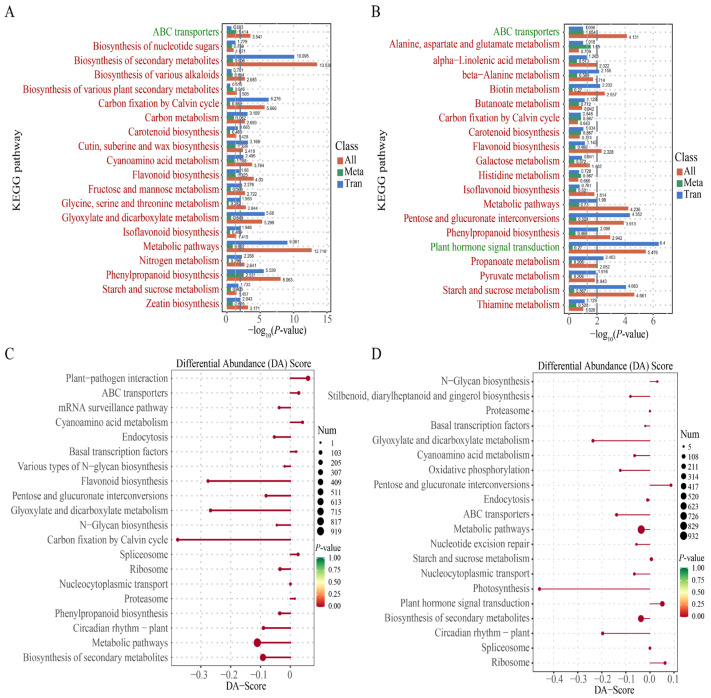
Integrated transcriptomic and metabolomic analysis of JBX1 and XBX4 under heat stress. (**A**,**B**) KEGG pathway enrichment analysis of DEGs and DAMs in JBX1 (**A**) and XBX4 (**B**). (**C**,**D**) DA score analysis of metabolic pathways in JBX1 (**C**) and XBX4 (**D**).

To further investigate the coordinated regulation of gene expression and metabolite accumulation in *P. ternata* under 40 °C heat stress, an integrated KEGG enrichment analysis and differential abundance (DA) score analysis were performed for JBX1 and XBX4. KEGG analysis revealed distinct regulatory focuses between JBX1 and XBX4 (Figure 8A,B). In JBX1, the highly enriched pathways in both transcripts and metabolites were “phenylpropanoid biosynthesis” (−log_10_*P* = 8.063), “flavonoid biosynthesis” (−log_10_*P* = 4.03), “carbon metabolism” (−log_10_*P* = 2.693) and “cutin suberin and wax biosynthesis” (−log_10_*P* = 2.418). The “carbon fixation by Calvin cycle“ pathway was primarily enriched at the transcriptomic level, whereas “ABC transporters” exhibited predominant enrichment at the metabolomic level (Figure 8A). Unlike JBX1, no single pathway in XBX4 exhibited co-significance at both the transcriptomic and metabolomic levels, highlighting a lack of coordination between gene expression and metabolic flux (Figure 8B). The molecular response of XBX4 was dominated by “plant hormone signal transduction”, which exhibited a high transcriptomic significance of 6.4. Pathways such as “starch and sucrose metabolism” (−log_10_*P* = 4.661) and “phenylpropanoid biosynthesis” (−log_10_*P* = 2.942) also showed significant enrichment. “ABC transporters” showed a high combined significance (−log_10_*P* = 4.131), driven largely by the metabolomic component (Figure 8B).

DA score analysis reflects the global metabolic trend in a pathway, with DA > 0 indicating upregulated metabolites, and DA < 0 suggesting downregulated metabolites. Several pathways exhibited positive DA scores in JBX1, indicating an overall activation or accumulation. These included “plant–pathogen interaction”, “ABC transporters”, “cyanoamino acid metabolism”, and “basal transcription factors” as well as protein-related pathways such as the “spliceosome”, “ribosome”, and “proteasome”. Conversely, pathways related to energy and secondary metabolism, such as “carbon fixation in photosynthetic organisms”, “glyoxylate and dicarboxylate metabolism”, “flavonoid biosynthesis”, and “phenylpropanoid biosynthesis”, showed negative DA scores, suggesting a high rate of consumption or suppression under extreme heat (Figure 8C). In XBX4, “pentose and glucuronate interconversions”, “plant hormone signal transduction”, “N-glycan biosynthesis”, and the “ribosome” pathways had positive DA scores. However, a majority of key pathways were significantly inhibited (negative DA scores), such as “ABC transporters”, “photosynthesis”, “circadian rhythm–plant”, and “glyoxylate and dicarboxylate metabolism” (Figure 8D). Notably, the DA score for “photosynthesis” in XBX4 reached an extremely low value of approximately −0.45, indicating a near-total metabolic collapse of the energy capture system. Based on the results of the integrated analysis, we further focused on pathways such as carbon metabolism, amino acid metabolism, the glutathione metabolic pathway, ABC transporters, and phenylpropanoid metabolism.

#### 2.7.1. Integrated Analysis of the Carbon Fixation Pathway

Pathways including the carbon fixation pathway (Calvin cycle), amino acid metabolism, glutathione metabolic pathway, ABC transporters, and phenylpropanoid metabolism were mapped to the transcriptomic and metabolomic data.

**Figure 9:**
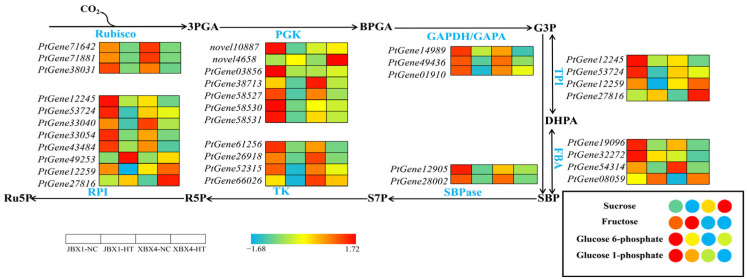
Integrated analysis of gene expression and metabolite accumulation in the carbon fixation pathway of JBX1 and XBX4 under heat stress. Schematic representation of the Calvin cycle showing key enzymatic steps. For each gene and metabolite, the four squares and circles represent relative transcript levels and metabolite abundances, respectively, in JBX1-NC, JBX1-HT, XBX4-NC, and XBX4-HT. Color scale indicates Z-score normalized expression (red: high; blue: low). Rubisco: ribulose-1,5-bisphosphate carboxylase/oxygenase; PGK: phosphoglycerate kinase; GAPDH/GAPA: glyceraldehyde-3-phosphate dehydrogenase; TPI: triose phosphate isomerase; FBA: fructose-1,6-bisphosphate aldolase; SBPase: sedoheptulose-1,7-bisphosphatase; TK: transketolase; RPI: ribose-5-phosphate isomerase; 3PGA: 3-phosphoglycerate; BPGA: 1,3-bisphosphoglycerate; G3P: glyceraldehyde-3-phosphate; DHPA: dihydroxyacetone phosphate; SBP: sedoheptulose-1,7-bisphosphate; S7P: sedoheptulose-7-phosphate; R5P: ribose-5-phosphate; Ru5P: ribulose-5-phosphate; G6P: glucose-6-phosphate; G1P: glucose-1-phosphate.

The carbon fixation pathway encompasses the core steps of the Calvin cycle, including carbon dioxide fixation, reduction, and the regeneration of ribulose-1,5-bisphosphate (RuBP). As shown in Figure 9, the majority of genes encoding core enzymes (e.g., ribulose-1,5-bisphosphate carboxylase/oxygenase (Rubisco), Phosphoglycerate kinase (PGK), 5-phosphate ribose isomerase (RPI), transketolase (TK), and triose phosphate isomerase (TPI)) exhibited significant downregulation in both JBX1 and XBX4 under HT. Compared with XBX4-NC, JBX1-NC exhibited significantly higher basal expression levels for numerous genes, notably those encoding PGK (*novel10887*, *PtGene03856*, *PtGene58530*, and *PtGene58531*), RPI (*PtGene12245* and *PtGene53724*), TPI (*PtGene12245*, *PtGene53724*), and fructose-1,6-bisphosphate aldolase (FBA) (*PtGene19096*, and *PtGene32272*). Similar to the transcriptional trends, the basal levels of key metabolites–including fructose, glucose-6-phosphate (G6P), and glucose-1-phosphate (G1P)–were significantly higher in JBX1-NC than in XBX4-NC. Conversely, a dramatic surge in sucrose content was observed in XBX4 following heat stress.

#### 2.7.2. Integrated Analysis of Nitrogen and Amino Acid Metabolism

**Figure 10:**
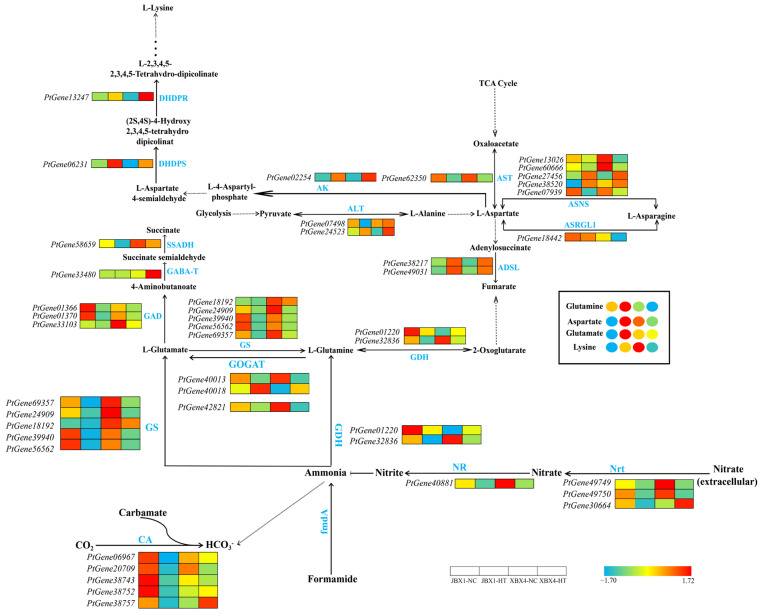
Integrated analysis of gene expression and metabolite accumulation in the nitrogen and amino acid metabolism pathway of JBX1 and XBX4 under heat stress. Schematic representation of the nitrogen and amino acid metabolism pathway showing key enzymatic steps. For each gene and metabolite, the four squares and circles represent relative transcript levels and metabolite abundances, respectively, in JBX1-NC, JBX1-HT, XBX4-NC, and XBX4-HT. Color scale indicates Z-score normalized expression (red: high; blue: low). Nrt: nitrate transporter; NR: nitrate reductase; CA: carbonic anhydrase; GS: glutamine synthetase; GOGAT: glutamine oxoglutarate aminotransferase; GDH: glutamate dehydrogenase; GAD: glutamate decarboxylase; AST: aspartate aminotransferase; AK: aspartate kinase; DHDPS: dihydrodipicolinate synthase; ASNS: asparagine synthetase; ADSL: adenylosuccinate lyase; DHDPR: dihydrodipicolinate reductase; fmdA: formamidase; ALT: alanine aminotransferase; ASRGL1: asparaginase-like protein 1; SSADH: succinate semialdehyde dehydrogenase; GABA-T: 4-aminobutyrate aminotransferase.

Under NC, the expression levels of several key enzymatic genes, including nitrate transporter (NRT), nitrate reductase (NR), and glutamine synthetase (GS), were significantly higher in XBX4 than in JBX1, with the exception of carbonic anhydrase (CA), which showed higher expression in JBX1. At the transcriptomic level, JBX1 and XBX4 displayed similar patterns, with most genes encoding core enzymes—including GS, glutamine oxoglutarate aminotransferase (GOGAT), glutamate dehydrogenase (GDH), NR, NRT, CA, glutamate decarboxylase (GAD), and aspartate aminotransferase (AST)—being significantly suppressed following heat treatment. However, the expression of genes related to aspartate kinase (AK) and dihydrodipicolinate synthase (DHDPS) was uniquely upregulated. At the metabolomic level, JBX1 and XBX4 displayed diametrically opposed metabolic trends under heat stress: the levels of glutamate (Glu), glutamine (Gln), aspartate (Asp), and lysine (Lys) in JBX1 showed a significant upward trend, while conversely, XBX4 exhibited a sharp decline in these core metabolites (Figure 10).

#### 2.7.3. Integrated Analysis of Phenylpropanoid Biosynthesis Pathway

**Figure 11:**
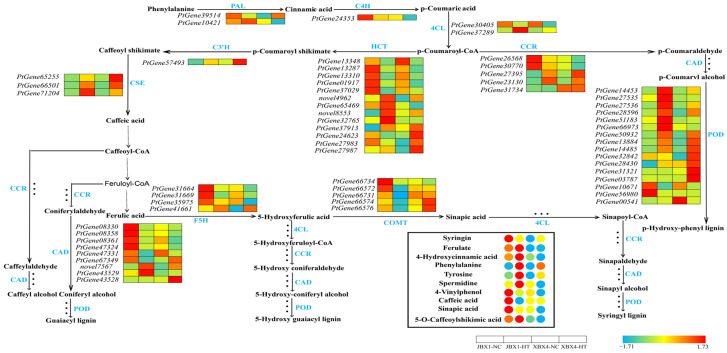
Integrated analysis of gene expression and metabolite accumulation in the phenylpropanoid biosynthesis pathway of JBX1 and XBX4 under heat stress. Schematic representation of the phenylpropanoid biosynthesis pathway showing key enzymatic steps. For each gene and metabolite, the four squares and circles represent relative transcript levels and metabolite abundances, respectively, in JBX1-NC, JBX1-HT, XBX4-NC, and XBX4-HT. Color scale indicates Z-score normalized expression (red: high; blue: low). PAL, phenylalanine ammonia-lyase; C4H, cinnamate 4-hydroxylase; 4CL, 4-coumarate--CoA ligase; HCT, shikimate O-hydroxycinnamoyltransferase; C3’H, 5-O-(4-coumaroyl)-D-quinate 3′-monooxygenase; CSE, caffeoyl shikimate esterase; CCR, cinnamoyl-CoA reductase; F5H, ferulate 5-hydroxylase; COMT, caffeic acid 3-O-methyltransferase; CAD, cinnamyl alcohol dehydrogenase; POD, peroxidase.

The initial state analysis between JBX1 and XBX4 showed that core enzymatic genes, including the entry-point enzyme phenylalanine ammonia-lyase (PAL, *PtGene39514*), cinnamate 4-hydroxylase (C4H, *PtGene24353*), and cinnamyl alcohol dehydrogenase (CAD, *PtGene08330*), exhibited remarkably higher expression in JBX1 than in XBX4. The POD enzyme-encoding genes in both materials showed upregulated expression (Figure 11).

Key antioxidants and protective compounds—including syringin, ferulate, caffeic acid, sinapic acid, and 5-O-caffeoylshikimic acid—were maintained at their highest abundance in JBX1-NC. In stark contrast, XBX4-NC displayed a significantly depleted metabolic state across these compounds. Notably, the phenylalanine levels further increased in JBX1-HT, ensuring a steady supply of precursors for the phenylpropanoid flux (Figure 11).

#### 2.7.4. Integrated Analysis of Glutathione Metabolism

**Figure 12:**
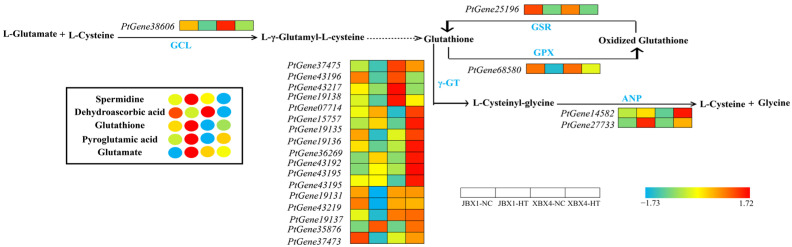
Integrated analysis of gene expression and metabolite accumulation in the glutathione metabolism pathway of JBX1 and XBX4 under heat stress. Schematic representation of glutathione metabolism pathway showing key enzymatic steps. For each gene and metabolite, the four squares and circles represent relative transcript levels and metabolite abundances, respectively, in JBX1-NC, JBX1-HT, XBX4-NC, and XBX4-HT. Color scale indicates Z-score normalized expression (red: high; blue: low). GCL, glutamate–cysteine ligase; GSR, glutathione reductase; GPX, glutathione peroxidase; *γ*-GT, *γ*-glutamyl transpeptidase; ANP, alanyl aminopeptidase.

As shown in Figure 12, both JBX1 and XBX4 showed an increased accumulation of glutathione (GSH) and pyroglutamic acid in response to heat stress; however, JBX1 exhibited a significantly higher accumulation efficiency. Interestingly, this metabolic advantage in JBX1 was not reflected at the transcriptional level. Conversely, the expression of genes encoding key enzymes, such as glutamate–cysteine ligase (GCL) (*PtGene38606*) and γ-glutamyl transpeptidase (γ-GT) (*PtGene37475*, *PtGene43196*, *PtGene43217*, *PtGene19138*, *PtGene37475*, *PtGene07714*, *PtGene15757*, *PtGene19135*, *PtGene19136*, *PtGene36269*, *PtGene43192*, *PtGene43195*), was more pronounced in XBX4 (Figure 12).

#### 2.7.5. Integrated Analysis of ABC Transporters

**Figure 13:**
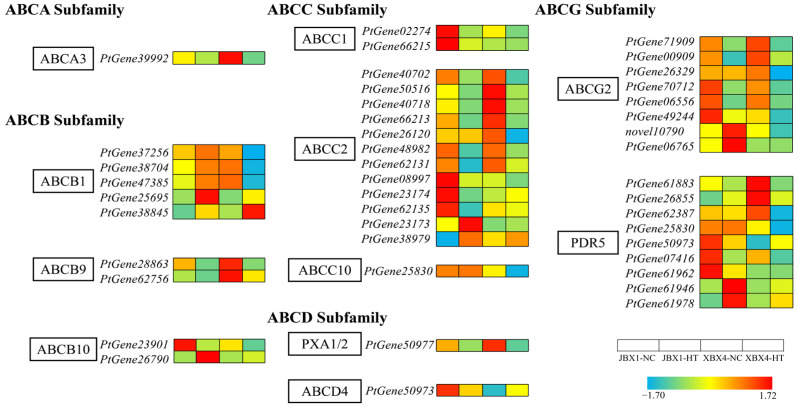
Integrated analysis of gene expression and metabolite accumulation in ABC transporters of JBX1 and XBX4 under heat stress. Schematic representation of ABC transporters showing key enzymatic steps. For each gene and metabolite, the four squares and circles represent relative transcript levels and metabolite abundances, respectively, in JBX1-NC, JBX1-HT, XBX4-NC, and XBX4-HT. Color scale indicates Z-score normalized expression (red: high; blue: low). ABC, ATP-binding cassette; ABCA/B/C/D/G, ATP-binding cassette subfamilies A/B/C/D/G; PDR, pleiotropic drug resistance; PXA1/2, peroxisomal ABC transporter 1/2.

We analyzed the expression profiles of the ABC transporter superfamily (Figure 13). Under NC conditions, the majority of genes in the ABCC subfamily (*ABCC1*, *ABCC2*, and *ABCC10*) exhibited significantly higher basal expression in JBX1 relative to XBX4. Similarly, the ABCG/PDR subfamily in JBX1 maintained consistently higher expression levels relative to XBX4 both before and after heat treatment. Furthermore, while JBX1 showed a coordinated upregulation of *ABCB1* genes and a partial induction of *ABCB10* members, XBX4 underwent a significant downregulation across these subfamilies. Collectively, these results underscore that JBX1 outperforms XBX4 in both the constitutive and induced regulation of ABC transporters.

#### 2.7.6. Network Analysis of Gene–Metabolite Associations in Core Pathways

**Figure 14:**
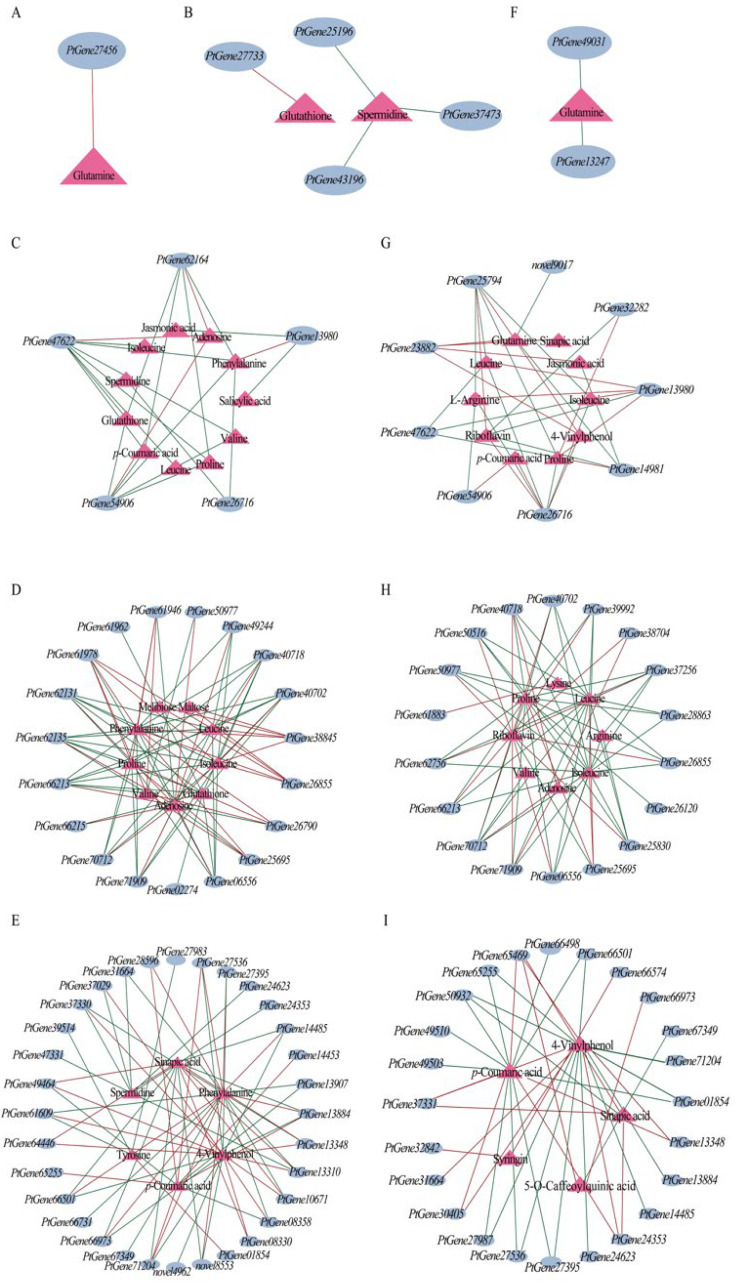
Gene–metabolite correlation network analysis of core metabolic pathways in JBX1 and XBX4 under heat stress. Networks encompass amino acid metabolism, glutathione metabolism, ABC transporters, and phenylpropanoid biosynthesis. (**A**) Amino acid metabolism in JBX1. (**B**) Glutathione metabolism in JBX1. (**C**) Correlation network between candidate thermotolerance-related genes (*TWA1*, *H2A.Z*, *PhyB*, *HSF*, *bZIP28*, and *HSPs*) and key DAMs in JBX1. (**D**) ABC transporter pathway in JBX1. (**E**) Phenylpropanoid biosynthesis in JBX1. (**F**) Amino acid metabolism in XBX4. (**G**) Correlation network between candidate thermotolerance-related genes (*TWA1*, *H2A.Z*, *PhyB*, *HSF*, *bZIP28*, and *HSPs*) and key DAMs in XBX4. (**H**) ABC transporter pathway in XBX4. (**I**) Phenylpropanoid biosynthesis in XBX4. Blue ellipses denote differentially expressed genes (DEGs); purple triangles denote differentially accumulated metabolites (DAMs). Edge colors indicate correlation direction: red for positive correlation (*r* ≥ 0.99), blue for negative correlation (*r* ≤ −0.99).

A co-expression network analysis (with a filtering criterion of |r| > 0.99) was performed for DEGs and DAMs involved in several core metabolic pathways, including the carbon fixation, amino acid metabolism, glutathione metabolic, ABC transporter, and phenylpropanoid metabolism pathways (Figure 14).

In the amino acid metabolism pathway, one gene (*PtGene27456*) in JBX1 showed a strong positive correlation with glutamine; in contrast, the related genes (*PtGene49031*, *PtGene13247*) in XBX4 both exhibited strong negative correlations, reflecting a polarity reversal in the thermal response between two materials (Figure 14A,F). The transcript levels of *PtGene27456*, *PtGene49031*, and *PtGene13247*—encoding the enzymes asparagine synthetase (ASNS), adenylosuccinate lyase (ADSL), and dihydrodipicolinate reductase (DHDPR), respectively—were consistently induced by heat stress across both JBX1 and XBX4.

*PtGene27733*, which encodes alanyl aminopeptidase (ANP), was upregulated in JBX1 and exhibited a strong positive correlation with glutathione (Figure 14B). The other three genes showed a significant negative correlation with spermidine, among which *PtGene25196* encodes glutathione reductase (GSR), and *PtGene37473* and *PtGene43196* encode γ-GT (Figure 14B). Notably, the expression levels of these three candidate genes were significantly downregulated in JBX1 under heat stress. In contrast, no highly correlated nodes were identified within the glutathione metabolic pathway of XBX4. These results provide a molecular basis for the observed enrichment of glutathione and spermidine in JBX1 following heat exposure, suggesting that JBX1 possesses a specialized capacity to directionally modulate the glutathione metabolic pathway to bolster its defense against thermal signatures.

In the ABC transporter pathway network, JBX1 focused on 19 genes and 9 metabolites; XBX4 focused on 18 genes and 8 metabolites (Figure 14D,H). Phenylalanine, proline, and adenosine occupied central hub positions in JBX1; *PtGene26855* (*PDR5*) and *PtGene38845* (*ABCB1*) were positively correlated with these metabolites, whereas genes such as *PtGene62131* (*ABCC2*) and *PtGene62135* (*ABCC2*) showed significant negative correlations (Figure 14D). Unlike JBX1, the hub metabolites in XBX4 were riboflavin, proline, leucine, and isoleucine. Among these, riboflavin was positively correlated with most genes, whereas proline, leucine, and isoleucine exhibited predominantly negative correlations (Figure 14H).

The phenylpropanoid biosynthetic network in JBX1 exhibited a more intricate and coordinated topological architecture, encompassing 32 differentially expressed genes. Within this network, phenylalanine and 4-vinylphenol were identified as central hub nodes, with the connectivity predominantly characterized by significant positive correlations, suggesting a highly synchronized mobilization of defensive metabolic flux (Figure 14E). In contrast, the network in XBX4 was markedly contracted, containing only 24 genes. The associations in XBX4 were primarily concentrated around the 4-vinylphenol and *p*-coumaric acid nodes, yet they exhibited a predominantly negative regulatory polarity (Figure 14I). Overall, the reduced scale, complexity, and integration of the XBX4 network underscore the inherent limitations of this sensitive germplasm in orchestrating secondary metabolic defenses under extreme thermal stress.

To further elucidate the systemic regulatory mechanisms, co-expression networks were constructed between candidate genes (*TWA1*, *H2A.Z*, *phyB*, *HSF*, *bZIP28*, and *HSPs*) and core DAMs associated with carbon fixation, amino acid metabolism, glutathione metabolism, ABC transporters, and phenylpropanoid biosynthesis. Our analysis revealed that while *H2A.Z*, *phyB*, and *bZIP28* showed no significant correlation with these metabolic pathways in JBX1 and XBX4, the regulatory hotspots were predominantly concentrated within the *PtTWA1* gene and the *HSF* transcription factor family. In JBX1, *PtTWA1* (*PtGene13980*) exhibited a robust positive correlation with phenylalanine, yet was inversely associated with jasmonic acid (JA) and salicylic acid (SA). Intriguingly, except for adenosine, the *HSF* gene family members in JBX1 displayed pervasive negative correlations with phenylalanine and other pivotal resistance-related metabolites, including proline, glutathione, spermidine, and valine (Figure 14C). This pattern suggests that *HSF*s may exert a negative regulatory influence on the accumulation of these metabolites during heat stress in JBX1. Conversely, in XBX4, the regulatory orientation of *PtTWA1* shifted significantly, showing positive correlations with amino acids such as proline and arginine, but a negative association with riboflavin. Furthermore, specific *HSF* members (e.g., *PtGene*23882, *PtGene*54906, and *PtGene*26716) in XBX4 exhibited a positive correlation with glutamate, sinapic acid, and *p*-coumaric acid—a stark contrast to the patterns observed in JBX1 (Figure 14G). Given that the expression of these HSF genes was downregulated under heat stress in both germplasms, this observed “polarity reversal” in transcript–metabolite correlations likely underscores the functional divergence between the two lines, partially explaining the preferential enrichment of core defensive metabolites in JBX1. Additionally, as primary executors of the heat stress response, HSP genes demonstrated an exceptionally high degree of network connectivity and integration in JBX1 (Supplementary Figure S5A). In contrast, the HSP–metabolite network in XBX4 was significantly sparser, indicating a functional contraction and systemic failure of the molecular chaperone system in the sensitive germplasm (Supplementary Figure S5B).

## 3. Discussion

### 3.1. Heat Stress Sensitivity in P. ternata

*P. ternata* as a typical sciophyte adapted to mild and humid understory environments (15–25 °C), the genetic background of which dictates its heat sensitivity. Ambient temperatures exceeding 30 °C can trigger “sprout tumble”, manifested by rapid chlorosis, wilting, and necrosis of the aerial tissues. Our multi-omics analysis revealed that extreme heat (40 °C) triggers a systemic collapse of metabolic homeostasis, rather than a coordinated stress response. The concurrent suppression of primary carbon and nitrogen assimilation pathways suggests a fundamental paralysis of energy production, while the broad repression of phenylpropanoid and flavonoid biosynthesis indicates a failure to maintain biochemical and structural defense barriers. This metabolic breakdown is consistent with the observed decline in *Pn* and the elevation of MDA, collectively pointing to an irreversible loss of cellular integrity [26,27].

From an evolutionary ecology perspective, “sprout tumble” serves as an adaptive heat avoidance strategy evolved by *P. ternata* to mitigate irreversible physiological damage. High temperature and drought are both critical abiotic stress factors that induce “sprout tumble” in *P. ternata*, yet the modalities of cellular demise induced by these stressors are fundamentally distinct. *P. ternata* perceives thermal stress in a basipetal (top-down) fashion; previous histological assessments revealed that heat-induced collapse is characterized by the mechanical rupture of cell walls and plasma membranes, followed by cytoplasmic efflux. These features are hallmarks of necrosis, classifying heat stress as a form of direct, acute injury [16]. In contrast, drought-induced collapse is driven by programmed cell death (PCD). This process is marked by chromatin condensation and nuclear pyknosis, where the nuclei undergo marginalization into an irregular shape. Consequently, a prolonged period of drought stress is typically required to trigger “sprout tumble” [7]. Overall, heat stress imposes a more immediate and violent physiological insult, necessitating a higher threshold of innate thermotolerance. Our study underscores a pronounced metabolic vulnerability in XBX4, which exhibited catastrophic metabolite exhaustion under thermal heat stress. We propose that this inherent lack of metabolic plasticity is the primary driver of its susceptibility, rendering the plant incapable of effectively buffering against abrupt heat shocks.

### 3.2. Enhanced Photosynthetic and Antioxidant Systems in JBX1

Heat stress disrupts cellular redox homeostasis by inducing the generation of ROS, such as H_2_O_2_, which leads to membrane lipid peroxidation (measured as MDA) [31,32]. To cope with ROS bursts, plants have evolved adaptive mechanisms including enzymatic and non-enzymatic antioxidant systems, among which SOD, CAT, and APX are key ROS-scavenging enzymes [33]. Studies have shown that heat-tolerant genotypes generally maintain lower levels of oxidative damage under high temperature, as reported in chickpea [34], mungbean [35], and lentil [36]. These results are consistent with our findings that JBX1 exhibited lower H_2_O_2_ and MDA levels, indicating reduced membrane lipid peroxidation and oxidative damage. Meanwhile, heat-tolerant genotypes across various crop species, including tomato, wheat, and lentil, consistently exhibit a more robust antioxidant enzyme system under heat stress, involving key enzymes such as SOD, CAT, POD, APX, and glutathione reductase (GR) [36,37,38]. These findings are consistent with our observation that JBX1 possesses higher basal activities of CAT and SOD under non-stressed conditions, contributing to its superior heat tolerance. Interestingly, after heat stress, the sensitive genotype XBX4 exhibited a more pronounced induction of antioxidant enzymes, particularly CAT and APX, compared to JBX1. This phenomenon aligns with observations in *Brassica juncea*, where the thermosensitive genotype NPJ-119 showed higher antioxidant enzyme activities under heat stress than the tolerant genotype NRCDR-02, yet still suffered greater oxidative damage [39]. These findings suggest that the absolute level of enzyme activity is not the sole determinant of heat tolerance; rather, the coordination of the antioxidant system, the timing of induction, and the overall efficiency in ROS scavenging are equally critical. This also implies that the contribution of specific antioxidant enzymes to thermotolerance may vary across species or even genotypes, highlighting the complexity of heat stress responses.

In the present study, JBX1 exhibited significantly higher chlorophyll content than XBX4 under both control and heat stress conditions. This characteristic is highly consistent with the “stay-green trait” reported in the literature. The stay-green trait refers to delayed leaf senescence, allowing plants to maintain photosynthetic activity under high temperature [40,41]. This trait has been associated with heat tolerance in multiple crops. For example, heat-tolerant genotypes in wheat, maize, and chickpea have been reported to maintain higher chlorophyll content or grain yield under heat stress [42,43,44,45]. These studies consistently demonstrate that the ability to maintain chlorophyll content is a common feature of heat-tolerant germplasms, and the performance of JBX1 is in full agreement with this pattern. Therefore, chlorophyll content can serve as an important indicator of heat tolerance in crops and is commonly used for screening heat-tolerant germplasm. This strategy is likely also applicable to the breeding of heat-tolerant *P. ternata* varieties.

Interestingly, although JBX1 exhibited a lower *Pn* than XBX4 under control conditions, it displayed significantly greater photosynthetic resilience after heat stress, with a much smaller reduction in *Pn*. This phenomenon has also been reported in heat-tolerant germplasms of various crops. Evidence from tomato, chickpea, and rice has demonstrated that heat-tolerant genotypes consistently exhibit greater photosynthetic stability under heat stress, maintaining higher photosynthetic rates or prolonged photosynthetic activity compared to their heat-sensitive counterparts [46,47,48]. These studies suggest that heat-tolerant germplasms generally possess greater photosynthetic system stability.

In conclusion, JBX1 can be attributed to the synergistic effects of a coordinated and efficient antioxidant defense system and photosynthetic system, which are core foundations of heat tolerance. Traits such as stay-green characteristic, photosynthetic rate, and antioxidant enzyme system can serve as effective indicators for screening heat-tolerant genotypes, and these parameters may also be applied in the breeding of heat-resistant varieties in *P. ternata*. Furthermore, our findings offer practical guidance for cultivation management. Given that extreme heat events are projected to increase in frequency, agronomic interventions such as shading cultivation could be strategically deployed during the most heat-sensitive developmental windows. These measures may effectively lower the ambient temperature within the *P. ternata* canopy, thereby delaying the onset of sprout tumble.

### 3.3. Stronger Basal Thermotolerance and Systemic Robustness in JBX1 than XBX4

Basal thermotolerance refers to the inherent ability of plants to withstand sudden lethal high temperatures without prior heat acclimation. In this study, compared with XBX4, JBX1 exhibited superior basal thermotolerance, with its advantage stemming from the synergistic enhancement of basal reserves and dynamic metabolic regulation. Structurally, JBX1 possesses greater leaf thickness, higher chloroplast content, and enhanced activity of antioxidant enzymes (SOD/CAT), maintaining carbon fixation capacity and stay-green traits. Metabolically, JBX1 specifically accumulates amino acids such as Glu, Gln, Asp, and Lys, which serve both as osmolytes for osmotic regulation and as precursors for heat-responsive protein synthesis. The detoxification system relies on high basal GSH reserves and the coordinated action of ABCC/ABCG transporters to clear ROS and transport toxic substances. In contrast, XBX4 experiences metabolic collapse due to impaired carbon transport (sucrose accumulation), accelerating the degradation of the photosynthetic system. JBX1′s “high basal expression-strong induced response” dual-regulation mode constitutes its core thermotolerance mechanism, providing key insights for plant thermotolerance research.

JBX1 demonstrated significantly enhanced systemic robustness compared to XBX4, as reflected in the coordinated architecture of its regulatory networks across core metabolic pathways. In the phenylpropanoid biosynthesis network, JBX1 exhibited a positively correlated regulatory cluster centered on phenylalanine and 4-vinylphenol, indicating an effective coordination of defensive metabolic flux. In contrast, the corresponding network in XBX4 was markedly contracted and characterized by predominantly negative regulatory associations, suggesting a compromised ability to mount a coherent secondary metabolic defense under thermal stress. Furthermore, JBX1 displayed directional regulatory capacity, exemplified by the positive coupling between specific genes and key protective metabolites—such as glutamine and glutathione—ensuring the targeted accumulation of critical defense substances. XBX4, however, exhibited a reversal of this regulatory polarity, with gene–metabolite correlations often trending negative, a pattern consistent with the observed metabolic dysregulation and pathological accumulation of sucrose. HSPs function as molecular chaperones to refold or stabilize denatured proteins, thereby mitigating cellular damage [49,50]. Consistent with these network-level differences, the HSP chaperone system in JBX1 was highly integrated and robust, whereas the HSP network in XBX4 appeared sparse and functionally contracted, likely exacerbating its vulnerability to heat-induced proteotoxic damage.

In *Arabidopsis* as a thermosensor, TWA1 directly activates the expression of the master heat-responsive transcription factor HSFA2 upon sensing elevated temperatures [51]. HSFA2, acting as a pivotal molecular switch, subsequently triggers the massive transcription of downstream HSPs [52]. The transcriptional induction of the *PtTWA1*-*PtHSFA2* axis in JBX1 was significantly more robust than in XBX4 under heat stress. Our findings revealed a striking genotype-specific regulatory polarity reversal of HSF transcription factors on metabolic networks under heat stress. In JBX1, *HSFs* negatively correlate with defense metabolites (phenylalanine, proline, glutathione), while *PtTWA1* positively associates with phenylalanine but negatively with JA/SA. Considering that *PtTWA1* is upregulated under heat stress whereas HSF genes are downregulated, this suggests that in JBX1, the *TWA1–HSF* axis potentially activates secondary metabolic pathways such as phenylpropanoid and spermidine biosynthesis. In contrast, in XBX4, *HSFs* are positively linked to amino acids and secondary metabolites.

JBX1’s systemic robustness advantage stems not only from the optimization of individual metabolic pathways but also from the coordinated integration of multi-level regulatory networks, forming a “high basal expression-strong induced response” dual-layer defense strategy. This system-level stability enables JBX1 to maintain physiological homeostasis and effectively activate defense mechanisms under heat stress. In contrast to the efficient mobilization in JBX1, XBX4 exhibited characteristics typical of “metabolic collapse”. The inability to convert carbon sources into defensive amino acids or to export them from heat-damaged leaves triggers severe negative feedback inhibition, accelerating the disintegration of the photosynthetic apparatus and ultimately leading to sprout tumble. This deficiency arises from dual inadequacy in both the basal thermotolerance and the response to heat stimuli.

We acknowledge that our study, which focused on two contrasting germplasms, has inherent limitations regarding the generalizability of the proposed regulatory signatures. While the contrasting phenotypes of JBX1 and XBX4 provided a powerful system for dissecting basal thermotolerance mechanisms, the robustness of specific candidates—such as the preferential accumulation of certain amino acids—as universal biomarkers for heat tolerance in *P. ternata* remains to be established. Future validation using a larger and genetically diverse panel of *P. ternata* accessions, as well as functional characterization in additional genetic backgrounds, will be essential to confirm the broader applicability of these findings.

## 4. Materials and Methods

### 4.1. Plant Materials and Heat High-Temperature Treatment

In this study, the heat-resistant (JBX1) and the heat-sensitive (XBX4) variety of *P. ternata* were used as experimental material. *Pinellia ternata* tubers (1 cm in diameter) were cultivated in a plant growth chamber with a 16 h/8 h light/dark photoperiod at 25 °C. To ensure consistency in the growth stage of the experimental materials, both JBX1 and XBX4 were selected from plants with uniform growth vigor at 10 days after emergence. To examine thermotolerance, they were directly exposed to high-temperature treatment at 40 °C with a light intensity of 8000 lux and relative humidity maintained at 60% for 72 h. Unfortunately, both germplasms exhibited “sprout tumble”, and therefore no recovery treatment was performed. At 8 h of heat treatment, leaf samples were collected from the plants for physiological index measurements, transcriptomic analysis, RT-qPCR analysis and metabolomic analysis.

### 4.2. Paraffin Sectioning of Leaves

The untreated leaves of XBX4 and JBX1 were collected and immediately fixed in Carnoy’s fluid (ethanol/glacial acetic acid, 3:1) at room temperature. Following fixation, the samples underwent dehydration through a graded ethanol series. The dehydrated leaves were then stained using the Safranin O-Fast Green protocol and cleared in xylene. Finally, the specimens were embedded in paraffin and sectioned into 10–15 µm thick slices using a Leica RM2135 microtome (Leica Microsystems, Wetzlar, Germany).

### 4.3. Measurement of Heat-Related Physiological Indexes

For the physiological and biochemical assays, three independent biological replicates were used. Each replicate consisted of leaf tissue pooled from five randomly selected plants grown in different pots within the same growth chamber. The content of MDA was determined following the protocol of Hodges et al. [53]. Briefly, 0.1 g of fresh sample was ground in 1 mL of 80% (*v*/*v*) ethanol. After centrifugation at 6000 rpm and 4 °C for 15 min, the supernatant was incubated with thiobarbituric acid (TBA) to generate the pink TBA–MDA complex. Absorbance was measured at 450, 532, and 600 nm using a UV–Vis spectrophotometer. The MDA content was calculated and normalized to fresh weight, expressed as nmol per gram fresh weight (nmol/g FW).

The content of H_2_O_2_ was assayed following the method of Zhao et al. [54]. Briefly, 0.5 g of fresh leaf sample was ground in 5 mL of acetone using a pre-chilled mortar. After centrifugation of the homogenate at 3000 rpm and 4 °C for 10 min, the supernatant was retained for H_2_O_2_ measurement. A reaction mixture was then prepared by combining 0.1 mL of 5% (*w*/*v*) titanium sulfate, 0.2 mL of concentrated ammonia, and 1 mL of the crude extract. This mixture was subjected to another centrifugation at 4 °C and 3000 rpm for 10 min. The obtained pellet was washed twice with 5 mL of acetone and subsequently dissolved in 5 mL of 2 M sulfuric acid. Absorbance was taken at 415 nm. The H_2_O_2_ content was calculated and expressed as nmol per gram fresh weight (nmol/g FW).

The accumulation of superoxide anion (O_2_^−^) in leaves was visualized in situ using a Nitroblue Tetrazolium (NBT) Staining Kit (Solarbio, Beijing, China) according to the manufacturer’s instructions. Briefly, fresh leaf samples from different treatments were immersed in the NBT staining working solution. To ensure thorough penetration of the stain into the mesophyll tissues, the samples were subjected to vacuum infiltration for 30 min and then incubated in the dark at 25 °C for 12 h. Subsequently, the stained leaves were transferred into decolorizing solution (80 °C, 40min) to completely remove the chlorophyll. The distribution of blue precipitates, representing O_2_^−^ accumulation, was then observed and photographed.

The activities of the antioxidant enzyme were assayed following the protocol of Zhao et al. [54]. Frozen leaf samples were homogenized at a ratio of 1:20 (*w*/*v*) in an extraction buffer composed of 50 mM PBS (pH 7.0), 1 mM EDTA, and 1% (*w*/*v*) polyvinylpyrrolidone. For APX activity measurement, the buffer was supplemented with 1 mM ascorbate. The homogenates were then centrifuged at 15,000× *g* and 4 °C for 20 min, and the resulting supernatants were immediately collected for enzyme activity assays. The activities of SOD, APX, and CAT were determined by measuring the respective decrease in absorbance at 560 nm, 290 nm, and 240 nm.

### 4.4. Chlorophyll Fluorescence Observation

The content of chlorophyll (Chl a + b, Chl a and Chl b) was measured by the acetone extraction method after 8 h of heat treatment. Chlorophyll content following 72 h of heat treatment was measured using a handheld SPAD meter (Konica Minolta, Japan). The *Pn*, gs, and Ci were measured by a CIRAS-3 portable photosynthesis system (PP Systems, Amesbury, MA, USA) after 8 h of heat treatment.

### 4.5. RNA-Seq

For the RNA-Seq analysis, JBX1 and XBX4 plants were subjected to heat stress at 40 °C for 8 h. Four treatment groups were established: JBX1-NC, JBX1-HT, XBX4-NC, and XBX4-HT. Each group included three independent biological replicates, yielding a total of 12 samples. All samples were sent to Wuhan Benna Technology Co., Ltd. (Wuhan, China) for library construction and sequencing. Total RNA was isolated using the TRIzol reagent (Invitrogen, Waltham, MA, USA) protocol. After passing quality control, RNA was enriched using oligo (dT) magnetic beads. The enriched mRNA was randomly fragmented into short pieces, followed by reverse transcription into cDNA. An A-tail was added, and sequencing adaptors were ligated. Library quality was assessed using an Agilent Bioanalyzer (Agilent Technologies, Santa Clara, CA, USA), and the libraries were accurately quantified with Qubit (Invitrogen, Waltham, MA, USA). Qualified libraries were sequenced on an Illumina HiSeq2500 platform (Illumina, San Diego, CA, USA), and clean reads were obtained after filtering out low-quality data.

### 4.6. Identification of Differentially Expressed Genes (DEGs) and Functional Analysis

Sequencing reads were aligned to the *P. ternata* reference genome (China National Center for Bioinformation (https://ngdc.cncb.ac.cn/gwh/Assembly/37791/show) (accessed on 8 June 2025)) using STAR (v2.7.10a, https://github.com/alexdobin/STAR accessed on 23 April 2026), followed by assembly with StringTie software (v2.2.1). The Gene expression levels were quantified using RSEM by counting the number of reads mapped to gene. These raw counts were then normalized to FPKM (fragments per kilobase per million mapped reads), where paired-end reads originating from the same fragment were counted as one fragment. DEGs were identified using DESeq2 (v1.34.0), with thresholds of |log_2_ (fold change)| ≥ 1 and false discovery rate (FDR) < 0.05. GO and KEGG pathway was conducted on DEGs using the clusterProfiler package (version 4.2.2). Significantly enriched terms were defined as those with an adjusted *p*-value ≤ 0.05 (Bonferroni correction for GO, and FDR for KEGG). Detailed parameters for each enriched pathway, including pathway size, gene coverage, and FDR-corrected *p*-values, are provided in Supplementary Table S3.

### 4.7. Metabolomic Profiling Analysis

For non-targeted metabolomics, JBX1 and XBX4 plants were subjected to heat stress at 40 °C for 8 h. Four treatment groups were established: JBX1-NC, JBX1-HT, XBX4-NC, and XBX4-HT. Each group included six independent biological replicates, yielding a total of 24 samples. All samples were sent to Wuhan Benna Technology Co., Ltd. for analysis. Briefly, freeze-dried sample powder (25 mg) was mixed with 1 mL of pre-chilled (−40 °C) extraction solvent (methanol/acetonitrile/water, 2:2:1, *v*/*v*/*v*) containing isotope-labelled internal standards. The mixture was homogenized at 35 Hz for 4 min, sonicated in an ice-water bath for 5 min (repeated three times), and then incubated at −40 °C for 1 h. After centrifugation at 12,000 rpm (13,800× *g*, 4 °C) for 15 min, the supernatant was filtered through a 0.22 μm membrane. An equal aliquot of each sample was pooled to prepare quality control (QC) samples. The analysis was performed on a Vanquish UHPLC system coupled to an Orbitrap Exploris 120 Mass Spectrometer (Thermo Fisher Scientific, Waltham, MA, USA). Separation was achieved on a Phenomenex Kinetex C18 column (2.1 mm × 50 mm, 2.6 μm) (Phenomenex Inc., Torrance, CA, USA). Mobile phase A was 0.01% acetic acid in water, and mobile phase B was isopropanol/acetonitrile (1:1, *v*/*v*). The flow rate was 0.2 mL/min, and the injection volume was 2 μL. The mass spectrometer was operated in both positive (spray voltage: 3.8 kV) and negative (spray voltage: −3.4 kV) ion modes. Sheath gas and auxiliary gas flow rates were 50 Arb and 15 Arb, respectively; capillary temperature was 320 °C. Data were acquired in information-dependent acquisition (IDA) mode. The raw data were converted to the mzXML format using ProteoWizard and processed with an in-house program, which was developed using R and based on XCMS, for feature detection, extraction, alignment, and integration. The R package and BiotreeDB (V3.0) (Benna Technology, Wuhan, China) were applied for metabolite identification. To minimize systematic bias and enhance the biological interpretability of the results, the raw metabolomic data were subjected to a series of preprocessing steps, including the filtration of features with excessive relative standard deviation (RSD), removal of features with missing values exceeding 50% in any single group or across all groups, imputation of remaining missing values using half of the minimum observed value, and normalization to the total ion current (TIC) of each sample. A complete list of all identified metabolites, along with their annotation details and relative abundances across samples, is provided in Supplementary Table S4.

To identify differentially accumulated metabolites (DAMs) between comparison groups, the variable importance in projection (VIP) values from the orthogonal partial least squares discriminant analysis (OPLS-DA) model were used. VIP > 1, and *p*-value < 0.05 from the Student’s *t*-test were considered DAMs. Furthermore, DAMs were subjected to pathway enrichment analysis using KEGG and MetaboAnalyst (https://www.metaboanalyst.ca/). A hypergeometric test was applied, and pathways with *p* < 0.05 were considered significantly enriched. Corresponding enrichment parameters for metabolomic pathways, including pathway size, metabolite coverage, and FDR-corrected *p*-values, are presented in Supplementary Table S5.

### 4.8. Integrated Transcriptome and Metabolome Analysis

Pearson correlation analysis was performed between the DEGs and DAMs for JBX1 and XBX4 using R (v4.1.2) to calculate the correlation coefficients. Gene–metabolite pairs with absolute correlation coefficients |r| ≥ 0.99 were selected for network visualization. Network graphs were generated using Cytoscape software (v3.10.4, https://cytoscape.org/), with targeted metabolic pathways highlighted for presentation.

The differential abundance (DA) score analysis was performed as described by A. Ari Hakimi et al. [55]. The DA score captures the tendency of a metabolic pathway to exhibit increased or decreased levels of metabolites relative to a control group. The DA score was calculated as the mean log_2_(FC) of all DAMs mapped to a given pathway. A positive DA score indicated overall metabolite upregulation (net accumulation), whereas a negative DA score suggested overall downregulation (net depletion or suppressed biosynthesis). DA score analysis was performed using in-house R scripts, and results were visualized as bar plots. By integrating DA scores with pathway enrichment information, the activation or inhibition status of metabolic pathways under heat stress was comprehensively evaluated.

### 4.9. RNA Extraction and RT-qPCR Analysis for Gene Expression

Plant total RNA was extracted using a plant RNA Extraction Kit (Omega Bio-Tek, Guangzhou, China). After verifying RNA integrity by electrophoresis, cDNA was synthesized with the HiScript II 1st Strand cDNA Synthesis Kit (Vazyme, Nanjing, China). RT-qPCR analysis was performed with 2× ChamQ Universal SYBR qPCR Master Mix (Vazyme, Nanjing, China) on a QIAquant 96 2plex Real-Time PCR System (QIAGEN, Hilden, Germany). *PtACTIN* mRNA was used as a control to normalize the expression data. Relative expression levels were calculated using the 2^−ΔΔCt^ method [56]. The primer sequences used in RT-qPCR are listed in Supplementary Table S2.

### 4.10. Statistical Analysis

In this study, each experiment was carried out on at least three biological repeats and three technical repetitions. The data used for the analyses were processed using GraphPad Prism (v10.3.0) software (San Diego, CA, USA). The means were compared by two-way ANOVA and Duncan’s multiple range test at the 5% significance level.

## 5. Conclusions

In summary, JBX1 possessed stronger basal thermotolerance, which was derived from the coordinated establishment of higher basal metabolic reserves and efficient dynamic metabolic reprogramming. These coordinated regulations help JBX1 overcome the inherent heat sensitivity of its species, allowing it to survive short bursts of extremely high temperatures. These findings provide a robust theoretical framework for the molecular breeding of heat-resistant *P. ternata* cultivars, offering strategies to mitigate the impact of transient extreme heat events in the context of global climate change.

## Data Availability

The original contributions presented in this study are included in the article/Supplementary Materials. Further inquiries can be directed to the corresponding author.

## Supplementary Materials

The following supporting information can be downloaded at: https://www.mdpi.com/article/10.3390/genes17050512/s1, Figure S1: Histochemical detection of superoxide anion (O_2_^.−^) by NBT staining in leaves of JBX1 and XBX4 under NC and HT (40 °C for 8 h). Figure S2: PCA and sample correlation analysis based on DEGs. (**A**) PCA score plot. (**B**) Heatmap illustrating the expression patterns of DEGs. (**C**) Correlation heat map of samples. (**D**) Boxplot showing the distribution of gene expression levels across samples. Figure S3: qRT-PCR validation of DEGs in JBX1 and XBX4 under heat stress. Figure S4: Metabolomic profiling of JBX1 and XBX4 under heat stress. (**A**) PCA plot of metabolite profiles. (**B**) Heatmap illustrating the changes in DAMs. Figure S5: Gene–metabolite correlation network analysis between *HSP* genes and core metabolites in JBX1 and XBX4 under heat stress. (**A**) Correlation network in JBX1. (**B**) Correlation network in XBX4. Supplementary Table S1: Transcriptome sequencing quality of 12 samples. Supplementary Table S2: The primers used for qRT-PCR in this study. Supplementary Table S3: Detailed pathway enrichment parameters for differentially expressed genes (DEGs) in JBX1 and XBX4 under heat stress. Supplementary Table S4: Complete list of identified metabolites in *Pinellia ternata* leaves under control (NC) and heat stress (HT) conditions. Supplementary Table S5: Detailed pathway enrichment parameters for DAMs in JBX1 and XBX4 under heat stress.
