# Supplementary figures and images for: Integrated Metabolomic and Transcriptomic Analyses Reveal the Differential Molecular Mechanisms Underlying Heat Stress Responses in Two Pinellia ternata Germplasms
Source: Genes (Basel). 2026 Apr 26;17(5):512. doi: 10.3390/genes17050512 (PMC13206143; doi:10.3390/genes17050512)

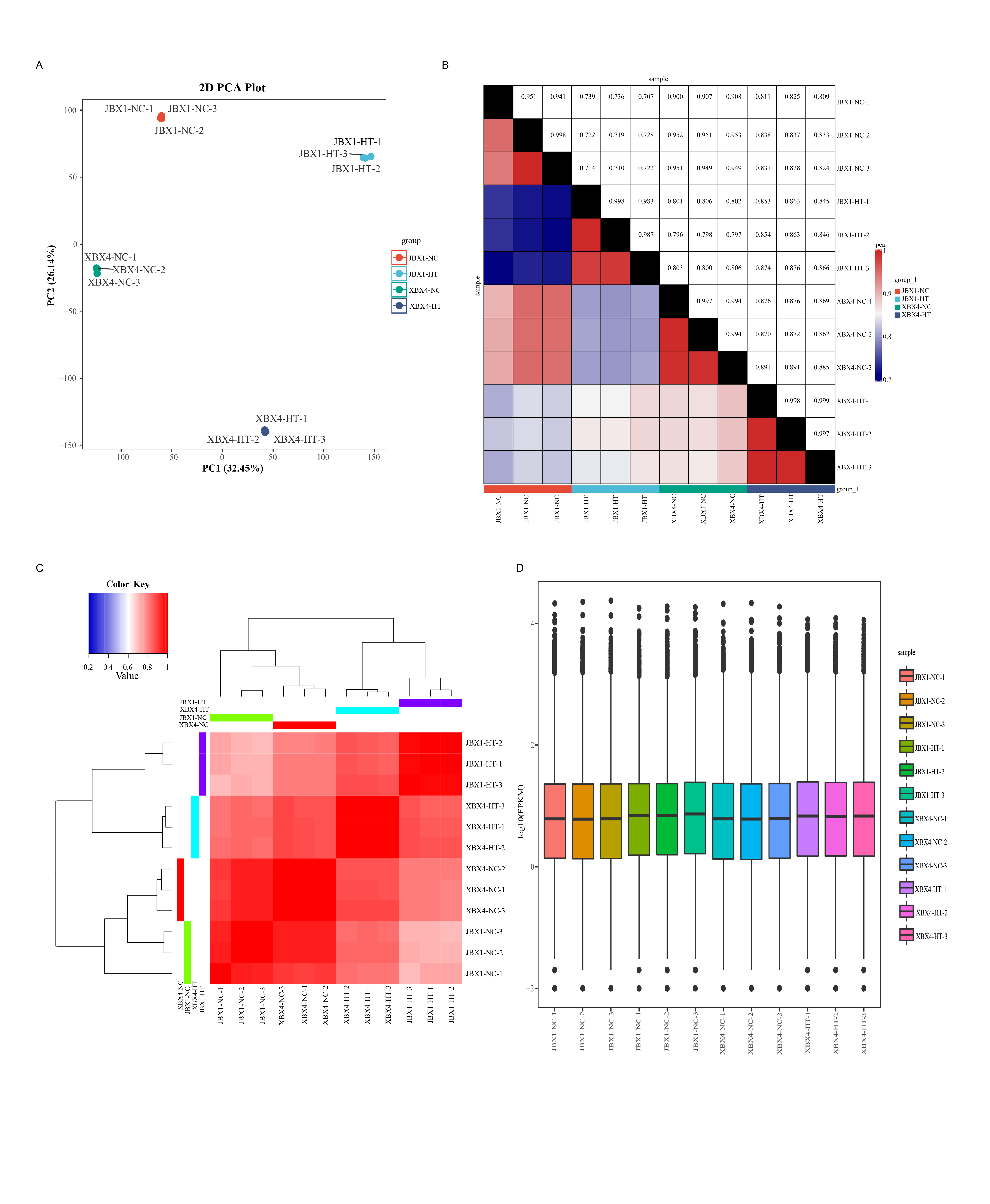

Supplement: Supplementary file 1 [file genes-17-00512-s001.zip › Supplementary Figure 2.png]

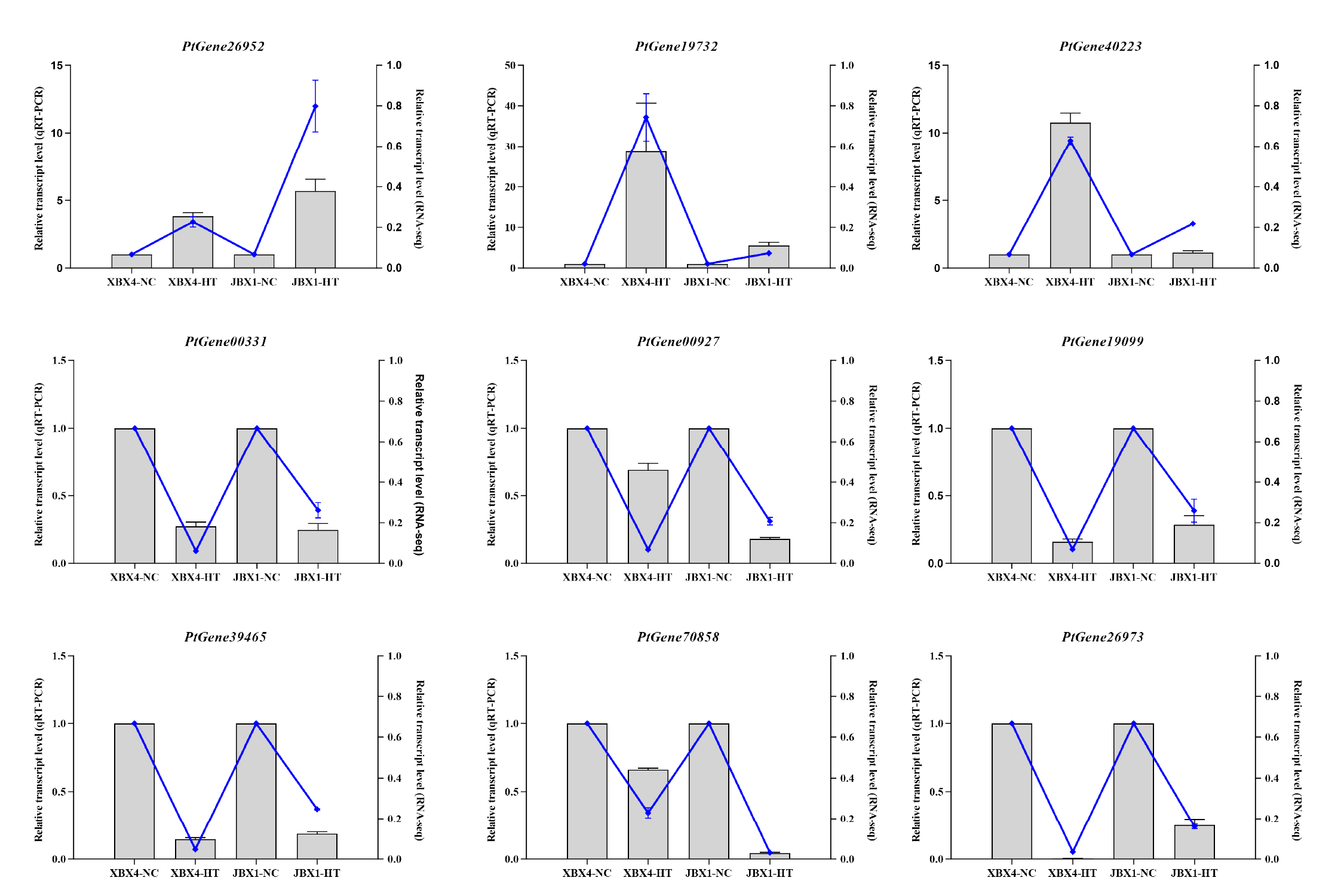

Supplement: Supplementary file 1 [file genes-17-00512-s001.zip › Supplementary Figure 3.png]

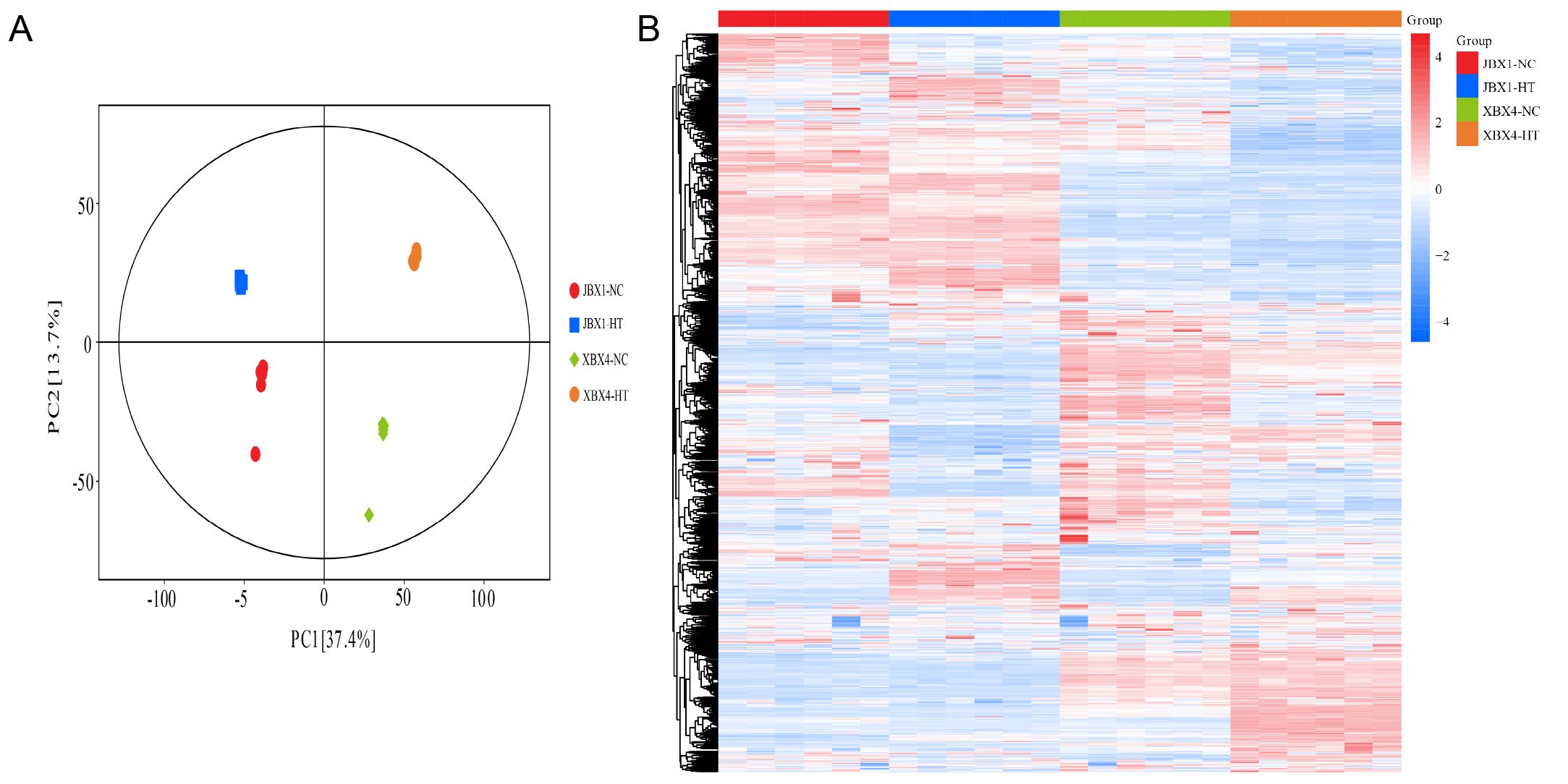

Supplement: Supplementary file 1 [file genes-17-00512-s001.zip › Supplementary Figure 4.png]
